# Identification and Characterization of circRNAs Responsive to Methyl Jasmonate in *Arabidopsis thaliana*

**DOI:** 10.3390/ijms21030792

**Published:** 2020-01-25

**Authors:** Jingjing Zhang, Ruiqi Liu, Yanfeng Zhu, Jiaxin Gong, Shuwei Yin, Peisen Sun, Hao Feng, Qi Wang, Shuaijing Zhao, Zhongyuan Wang, Guanglin Li

**Affiliations:** 1 Key Laboratory of Ministry of Education for Medicinal Plant Resource and Natural Pharmaceutical Chemistry, Shaanxi Normal University, Xi’an 710119, Shaanxi, China; zhangjjg@snnu.edu.cn (J.Z.); ruiqiliu@snnu.edu.cn (R.L.); gongjx@snnu.edu.cn (J.G.); yswdx_anwser@snnu.edu.cn (S.Y.); sps@snnu.edu.cn (P.S.); fenghao@snnu.edu.cn (H.F.); 2 College of Life Sciences, Shaanxi Normal University, Xi’an 710119, Shaanxi, China; hutian@snnu.edu.cn (Y.Z.); wq2017@snnu.edu.cn (Q.W.); jing347431@snnu.edu.cn (S.Z.); wangzhongyuan@snnu.edu.cn (Z.W.)

**Keywords:** circRNAs, jasmonic acid, *Arabidopsis thaliana*, GO enrichment, miRNA decoys

## Abstract

Circular RNAs (circRNAs) are endogenous noncoding RNAs with covalently closed continuous loop structures that are formed by 3′–5′ ligation during splicing. These molecules are involved in diverse physiological and developmental processes in eukaryotic cells. Jasmonic acid (JA) is a critical hormonal regulator of plant growth and defense. However, the roles of circRNAs in the JA regulatory network are unclear. In this study, we performed high-throughput sequencing of *Arabidopsis thaliana* at 24 h, 48 h, and 96 h after methyl JA (MeJA) treatment. A total of 8588 circRNAs, which were distributed on almost all chromosomes, were identified, and the majority of circRNAs had lengths between 200 and 800 bp. We identified 385 differentially expressed circRNAs (DEcircRNAs) by comparing data between MeJA-treated and untreated samples. Gene Ontology (GO) enrichment analysis of the host genes that produced the DEcircRNAs showed that the DEcircRNAs are mainly involved in response to stimulation and metabolism. Additionally, some DEcircRNAs were predicted to act as miRNA decoys. Eight DEcircRNAs were validated by qRT-PCR with divergent primers, and the junction sites of five DEcircRNAs were validated by PCR analysis and Sanger sequencing. Our results provide insight into the potential roles of circRNAs in the MeJA regulation network.

## 1. Introduction

Circular RNAs (circRNAs) are a type of noncoding RNA widely found in plants and animals. These molecules have a covalently closed continuous loop structure that typically derives from a backsplicing event in which the upstream 5’ splice acceptor is linked to the downstream 3’ splice donor [1]. Previously, circRNAs were believed to be produced by abnormal splicing [2]. Due to advances in high-throughput sequencing and bioinformatics analysis, many circRNAs have been identified in human, mice, rat, and other species [3,4,5]. Based on their splice positions in the genome, circRNAs can be divided into exonic circRNAs, intronic circRNAs, exonic-intronic circRNAs, and intergenic circRNAs [6]. Most circRNAs typically have low expression in eukaryotes, but a few are highly expressed in specific cell types, developmental stages or tissues, suggesting that they have key regulatory roles in various biological processes [7].

Increasing evidence indicates that circRNAs play important roles in regulating various biological processes in animals. One of the most common roles of circRNAs in animals is the regulation of gene expression in various biological processes, where they may act as microRNA (miRNA) decoys or sponges to sequester miRNAs [8,9,10,11,12]. Additionally, circRNAs interact with many different RNA binding proteins (RBPs) to function as protein sponges, to recruit proteins to specific locations or subcellular compartments, and to act as scaffolds to mediate complex formation between specific enzymes and substrates [13]. Furthermore, although most circRNAs are considered to be noncoding RNAs, some circRNAs undergo cap-free translation under certain conditions [14]. Recently, increasing numbers of circRNAs have been detected in *Arabidopsis thaliana* [15,16,17], maize [18,19,20], rice [15,21], tomato [22,23,24], wheat [25,26,27], and grape [28], indicating that circRNAs are widespread in plants. Plant circRNAs are as conserved and tissue specific as animal circRNAs, but their flanking introns do not contain as many repetitive elements and reverse complementary sequences as those in animals [15,16,19,29]. Recent studies have suggested that plant circRNAs play functional roles in developmental processes and stress responses, including the responses to drought, chilling injury, nutrient deficiency, and pathogen invasion [19,20,22,24,25,30]. Based on the functions of animal circRNAs, circRNA-miRNA-mRNA networks have been generated for plants; these networks suggest that plant circRNAs may act as miRNA sponges to regulate functional gene expression [23,25,31]. Additionally, plant circRNAs are involved in the regulation of their parental genes. For example, the expression of some exonic circRNAs and that of their host genes are significantly positively correlated in rice [15], and circRNAs in *Phyllostachys edulis* negatively regulate the expression levels of their cognate linear mRNAs, as evidenced from transcriptome sequencing [32]. Such findings suggest that the regulatory effects of circRNAs on the corresponding parental transcripts are diverse. Additionally, 1569 translated circRNAs have been detected in *A. thaliana* based on ribosome profile databases [33]. However, the mechanisms underlying their regulatory roles are poorly understood. To date, only one report elucidating the molecular mechanisms underlying the regulatory roles of plant circRNAs has been published, in which an exonic circRNA from *SEPALLATA3* in *A. thaliana* was shown to regulate the splicing of its parental mRNA through the formation of an R loop [34].

The phytohormone jasmonic acid (JA) and its derivatives, methyl ester (MeJA) and isoleucine conjugate (JA-Ile), are collectively referred to as jasmonates (JAs) [35]. JAs participate in the regulation of plant growth and developmental processes, such as leaf senescence and the development of roots, shoots, flowers, and seeds [36,37,38,39]. In particular, JAs can act as signaling molecules to regulate plant adaptation to abiotic stresses, such as wounding, ultraviolet radiation, and ozone, and biotic stresses, such as pathogen infection and herbivore attack. They do so by inducing the expression of JA-responsive genes and the accumulation of various secondary metabolites in plants [40,41,42,43]. However, whether circRNAs participate in plant responses to JA remains to be elucidated.

To explore the potential functions of circRNAs in JA signaling, we performed high-throughput sequencing of MeJA-treated *A. thaliana*. Differentially expressed circRNAs (DEcircRNAs) were identified and then verified by real-time quantitative PCR (qRT-PCR). In addition, DEcircRNAs as miRNA decoys were predicted. Our study reveals the potential functional roles of the circRNAs in the JA signaling network.

## 2. Results

### 2.1. Identification and Characterization of circRNAs

**Figure 1:**
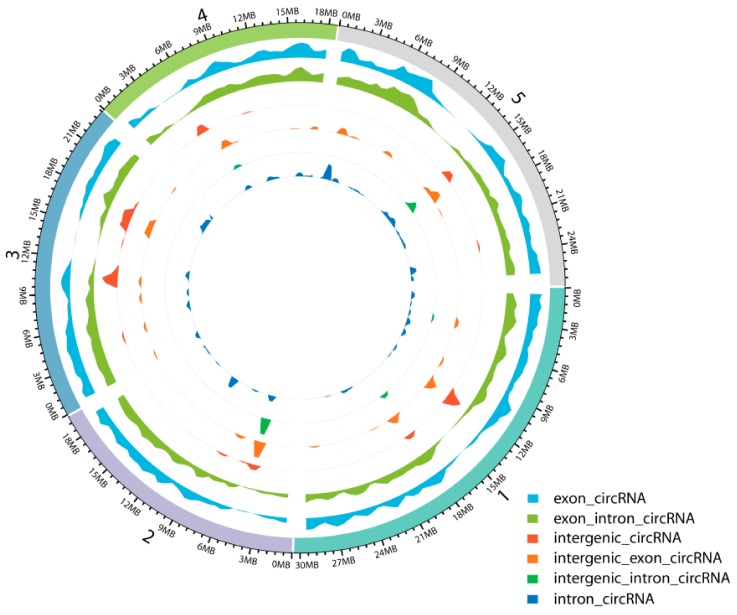
Density distribution of circRNAs on the chromosomes.

To identify circRNAs involved in JA-mediated signaling in *A. thaliana*, we constructed ribosomal (r)RNA-depleted RNA-Seq libraries for seedlings sampled at 24 h, 48 h, and 96 h after MeJA treatment and then conducted IlluminaHiSeq™ X-ten paired-end sequencing. Analysis of the circRNA sequencing data revealed that a total of 489,477,066 reads were generated from 12 samples of *A. thaliana* seedlings, in which the Guanine and Cytosine (GC) content was approximately 42%, and Q30 was greater than 93% (Table S1). We summarized the total numbers of circRNAs identified from the sequencing data by CIRI2, CIRCexplorer, and find_circ. A total of which 1526 circRNAs were predicted by CIRI2, 3410 circRNAs by CIRCexplorer, and 4564 circRNAs by find_circ; only 12 circRNAs were detected by all three algorithms (Figure S2) [5,44,45]. The expression levels of these 12 circRNAs are different; some are relatively high and some are relatively low, and most of them were expressed at more than one time points, while a few were expressed at only one time point. A total of 8588 unique circRNAs were obtained after the redundant circRNAs were removed by CD-HIT-EST [46,47]. The density distribution of these circRNAs is shown in Figure 1 and indicates that various chromosomal regions can produce circRNAs.

**Figure 2:**
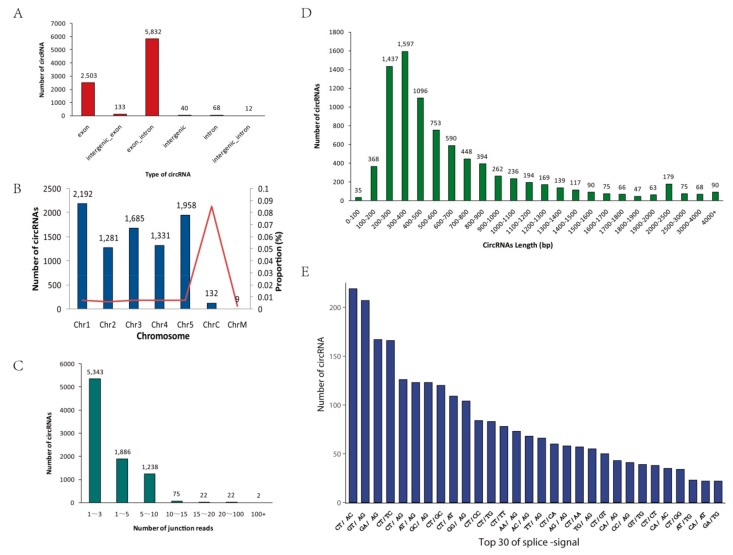
Characterization of circRNAs. (**A**) Classification of circRNAs. (**B**) The number and proportion of circRNAs detected in *A. thaliana* chromosomes. Proportion = (number of circRNAs on chromosome / chromosome length) × 100%. The bars represent the numbers of circRNAs, and the red line represents the proportion. (**C**) The number of junction-reads of circRNAs. (**D**) The length distribution of circRNAs. (**E**) Splicing signals of the circRNAs identified in our circRNA-seq datasets. The histogram shows the top 30 splicing signals of the circRNAs.

According to the start and end positions of circRNAs in the genome (splice site), these unique circRNAs were divided into the following six categories: exon, exon_intron, intergenic, intergenic_exon, intergenic_intron, and intron (Figure 2A). Most circRNAs originated from exons, and a few circRNAs originated from introns, indicating that regions that can produce mRNA are likely to produce circRNAs. The number of circRNAs on each chromosome was counted (Figure 2B). Most circRNAs were located on chromosomes 1 to 5; only a few circRNAs were found in chloroplasts or mitochondria, however, chloroplast chromosomes had the highest ratio of the number of circRNAs to chromosome length. Regarding the number of backspliced junction reads for circRNAs, most of the circRNAs in all samples contained fewer than 10 unique backspliced junction reads, indicating that the expression levels of most circRNAs were low (Figure 2C). In addition, the lengths of the candidate circRNAs were mainly distributed between 200 and 800 bp; a few circRNAs were longer than 1000 bp (Figure 2D). The splicing signal of circRNAs in animals is usually GT-AG; in contrast, the splicing signals at the splice sites of circRNAs in this study were diverse (Figure 2E).

**Figure 3:**
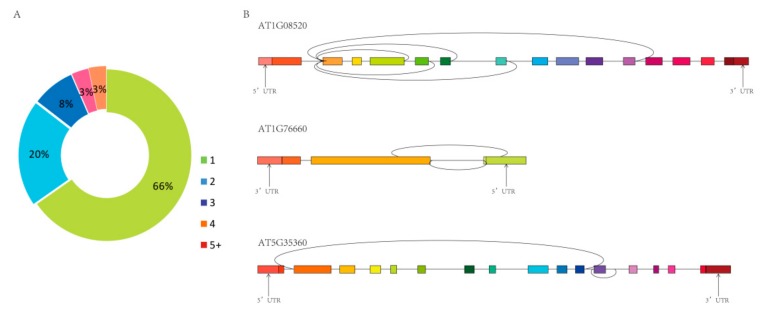
The number of circRNAs produced by the same host genes. (**A**) Percentage of circRNAs produced from the same host gene (7761 circRNAs from 4928 host genes). (**B**) Examples of three host genes that produce more than one circRNA.

Moreover, 7761 circRNAs were derived from 4928 host genes, of which 65.99% produced only one circRNA, and the remainder produced more than one circRNA (Figure 3A). The same host gene produced different circRNAs by alternative splicing, and these alternative circRNAs were unrelated to each other, or one contained the other, or one splice site was identical and the other splice site was different (Figure 3B).

### 2.2. Validation of circRNAs

**Figure 4:**
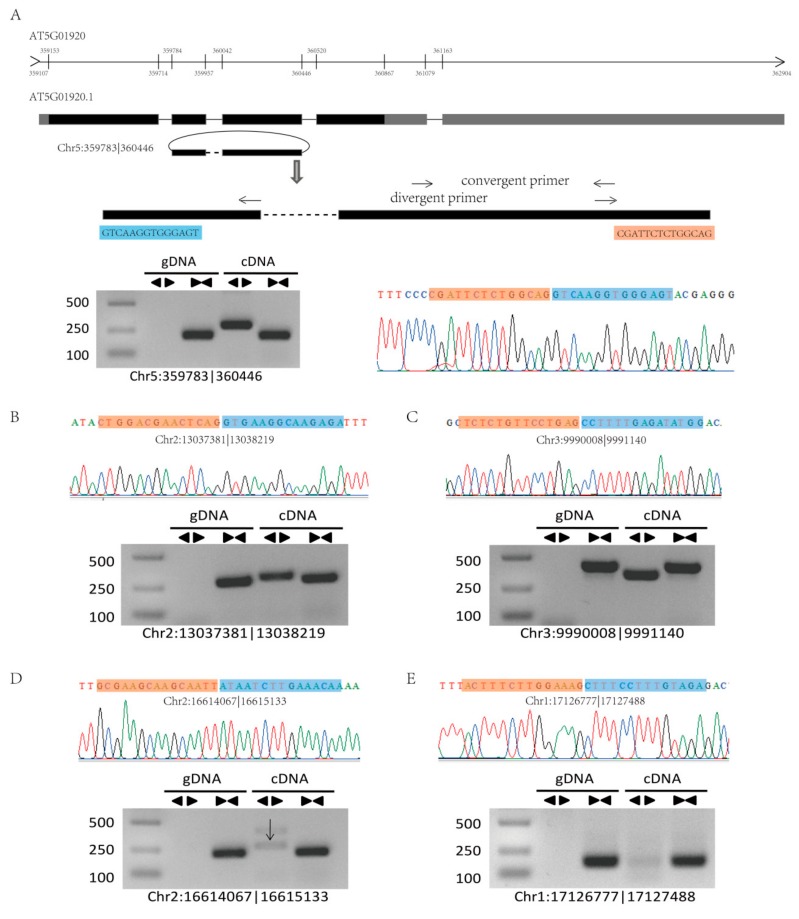
Validation of circRNAs by PCR and Sanger sequencing. (**A**) A circRNA (Chr5:359783|360446) exemplifying the validation strategy. According to the genomic loci, circRNA Chr5:359783|360446 was derived from the gene *AT5901920*. The two sets of arrows indicate two sets of amplification primers on the exon region, which were designed to confirm head-to-tail backsplicing by PCR and Sanger sequencing. (**B**–**E**) Validation of four circRNAs (Chr2:13037381|13038219, Chr3:9990008|9991140, Chr2:16614067|16615133, and Chr1:17126777|17127488) by PCR and Sanger sequencing. Divergent primers successfully amplified the circRNAs in cDNA but not those in gDNA. Convergent primers amplified the circRNAs in both cDNA and gDNA.

To confirm our identified circRNAs, we randomly selected six highly expressed circRNAs for experimental validation using polymerase chain reaction (PCR) and Sanger sequencing, and five circRNAs were successfully verified (Figure 4A,B and Figure S3). The structure of circRNAs differs from that of linear RNA, and thus, two sets of primers (convergent and divergent) and two templates (genomic DNA (gDNA) and complementary DNA (cDNA)) were used to verify the circRNAs (Figure 4). For example, circRNA Chr5:359783|360446 was located on the second to the third exon of the mRNA of the gene *AT5G01920* (Figure 4A). To validate this circRNA, we designed convergent and divergent primers on exon 2 and exon 3 and confirmed the sequence by Sanger sequencing after PCR amplification (Figure 4).

### 2.3. DEcircRNAs Induced by MeJA Treatment

**Figure 5:**
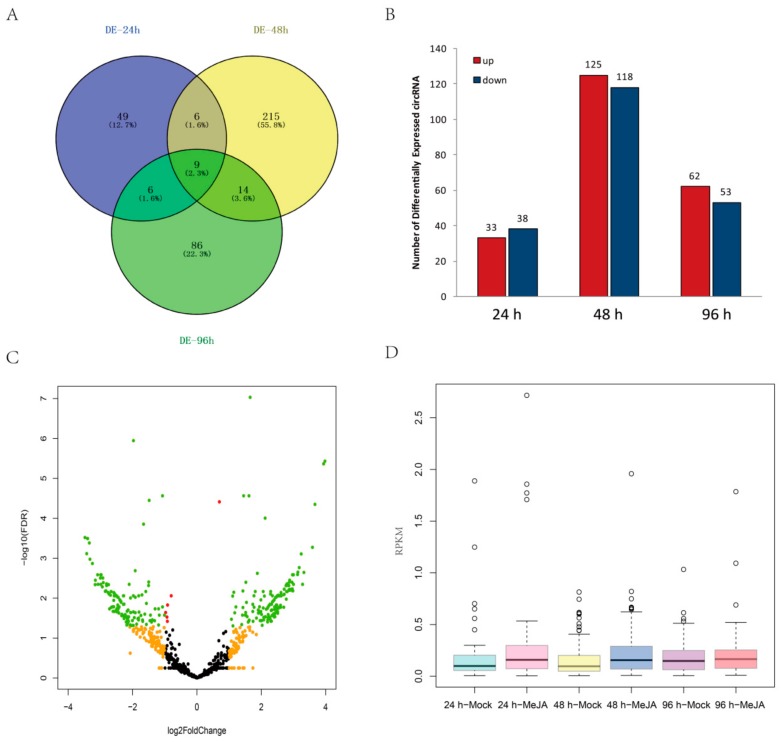
Analysis of DEcircRNAs. (**A**) Venn diagram of DEcircRNAs at three time points. (**B**) Regulation of DEcircRNAs at different time points. (**C**) Volcano map of DEcircRNAs between the control and MeJA treatments. Red nodes represent DEcircRNAs with false discovery rate (FDR) < 0.05, orange nodes represent DEcircRNAs with |log2FoldChange| > 1, and green nodes represent DEcircRNAs with FDR < 0.05 and |log2FoldChange| > 1. (**D**) Relative expression levels of DEcircRNAs after MeJA treatment.

The expression levels of some circRNAs in *A. thaliana* seedlings after MeJA treatment differed from those in controls. In total, 385 DEcircRNAs were detected between MeJA-treated and control seedlings among the three time points (24 h, 48 h, and 96 h) by using the circMeta R packages; among them, 70 circRNAs were differentially expressed at 24 h, 244 circRNAs at 48 h, and 115 circRNAs at 96 h (Figure 5A and Table S2). Moreover, nine circRNAs were differentially expressed at all three time points (Figure 5A). Among the DEcircRNAs, 33 were upregulated and 38 were downregulated at 24 h, 125 were upregulated and 118 were downregulated at 48 h, and 62 were upregulated and 53 were downregulated at 96 h (Figure 5B). In addition, a volcano map indicated that the fold changes of 379 DEcircRNAs were greater than 2 (|log2FoldChange| > 1) (Figure 5C). The expression levels of most DEcircRNAs were very low, with RPKM < 1, but the expression of most DEcircRNAs increased significantly at 24 h and 48 h after MeJA treatment (Figure 5D).

**Figure 6:**
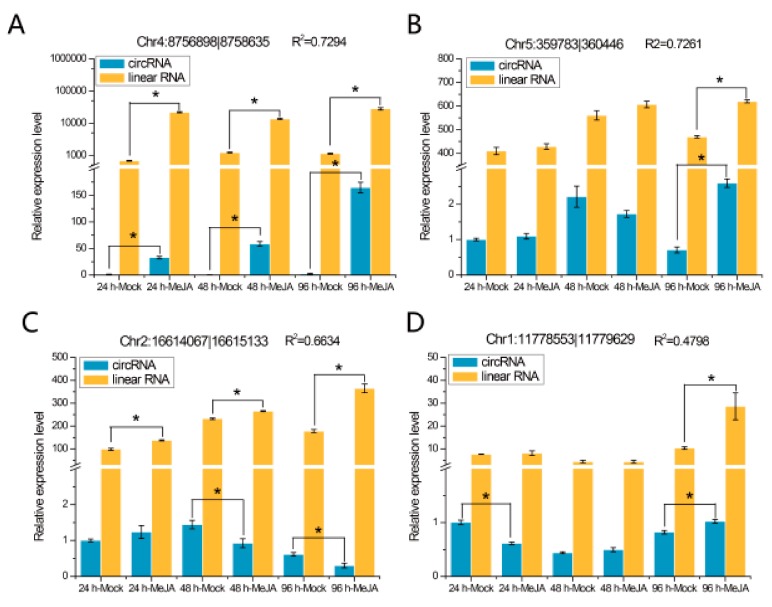

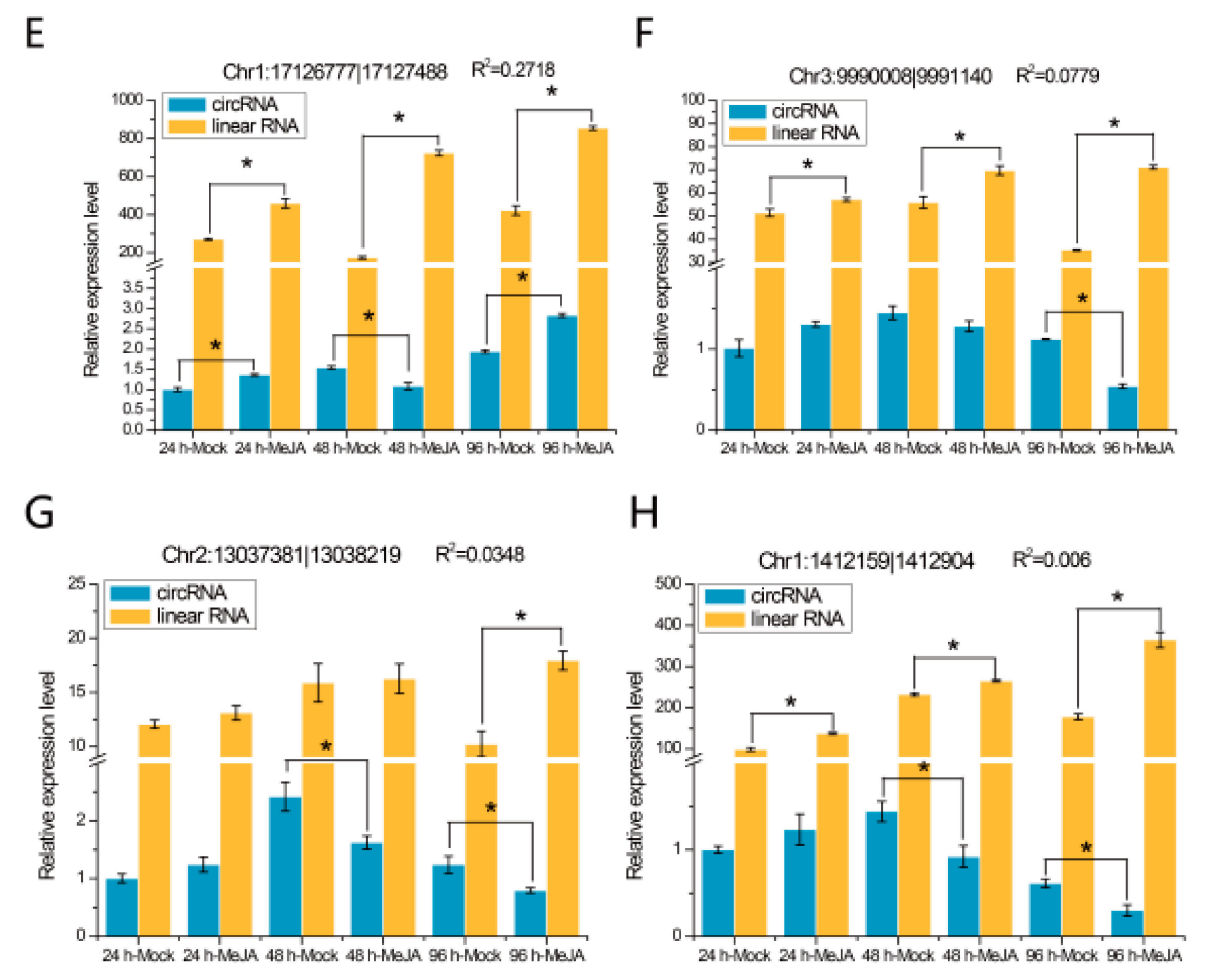
qRT-PCR validation of eight DEcircRNAs and their linear transcripts. (**A**) circRNA Chr4:8756898|8758635; (**B**) circRNA Chr5:359783|360446; (**C**) circRNA Chr2:16614067|16615133; (**D**) circRNA Chr1:11778553|11779629; (**E**) circRNA Chr1:17126777|17127488; (**F**) circRNA Chr3:9990008|9991140; (**G**) circRNA Chr2:13037381|13038219; (**H**) circRNA Chr1:1412159|1412905. Three replicates were established. R^2^ represents the correlation of expression level between the circRNAs and linear transcripts. Error bars represent the standard error of the mean. * *p*< 0.05, as determined by Tukey’s-b test.

The expression patterns of eight randomly selected DEcircRNAs were detected at different time points by quantitative real-time PCR (qRT-PCR), and the results are shown in Figure 6. We found that two of these eight DEcircRNAs, circRNA Chr4:8756898|8758635 (Figure 6A) and circRNA Chr1:17126777|17127488 (Figure 6E), were differentially expressed at all three time points, while the other six, such as circRNA Chr5:359783|360446 (Figure 6B) and circRNA Chr3:9990008|9991140 (Figure 6F), were differentially expressed at one or two time points. The expression levels of their mRNA were detected, and the results indicated that the expression levels of two circRNAs (Chr4:8756898|8758635 and Chr5:359783|360446) were related to the expression levels of their linear transcripts (Figure 6A,B; *R*^2^ > 0.7), whereas the expression levels of the other circRNAs were not correlated with those of their mRNA (Figure 6C–H).

### 2.4. Functional Prediction of DEcircRNAs by Gene Ontology (GO) Enrichment Analysis

**Figure 7:**
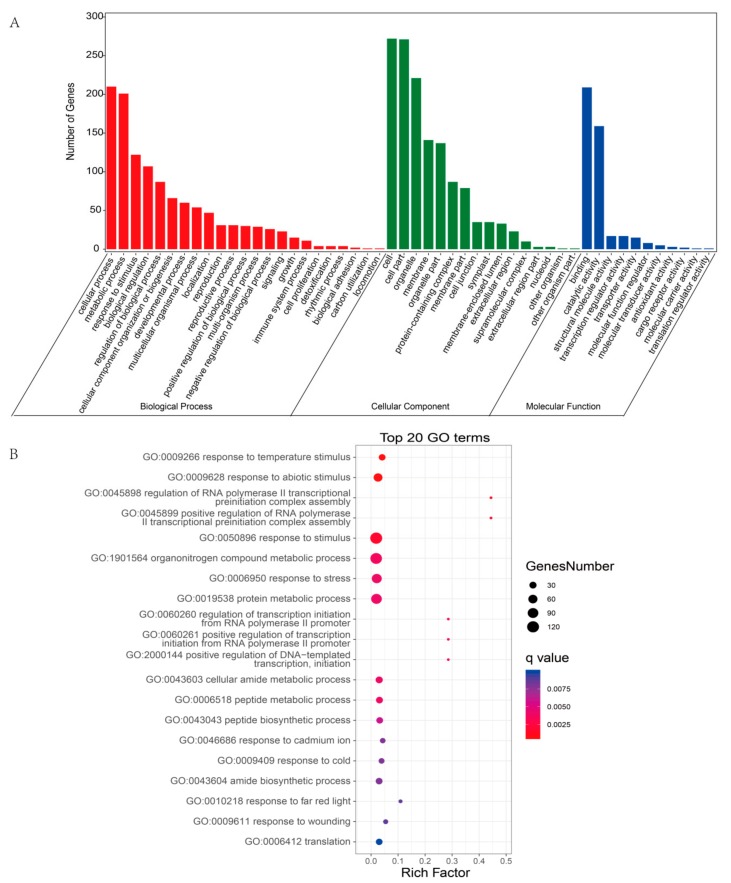
GO enrichment analysis of the host genes of DEcircRNAs. (**A**) The most enriched GO terms of the host genes of DEcircRNAs. (**B**) Top 20 GO enrichment categories of the host genes of DEcircRNAs.

The potential function of a circRNA may depend on the function of its host gene; thus, we analyzed the functions of DEcircRNA host genes [48]. The results of GO enrichment analysis showed that the host genes were involved in three categories: biological process (BP), cellular component (CC), and molecular function (MF) (Figure 7A and Table S6). In the BP category, most circRNAs were enriched in cellular process (GO:0009987), metabolic process (GO:0008152), response to stimulus (GO:0050896), biological regulation (GO:0065007), and developmental process (GO:0032502). In addition, the top 20 significant GO terms, including response to temperature stimulus (GO:0009266), response to abiotic stimulus (GO:0009628), response to stress (GO:0006950), and response to wounding (GO:0009611), are displayed in Figure 7B. These findings indicated that the host genes of the DEcircRNAs were associated with plant responses to stimuli. Thus, these DEcircRNAs might also be involved in the plant responses to stimulation.

### 2.5. Functional Prediction of DEcircRNAs Based on the circRNA-miRNA-mRNA Network

**Figure 8:**
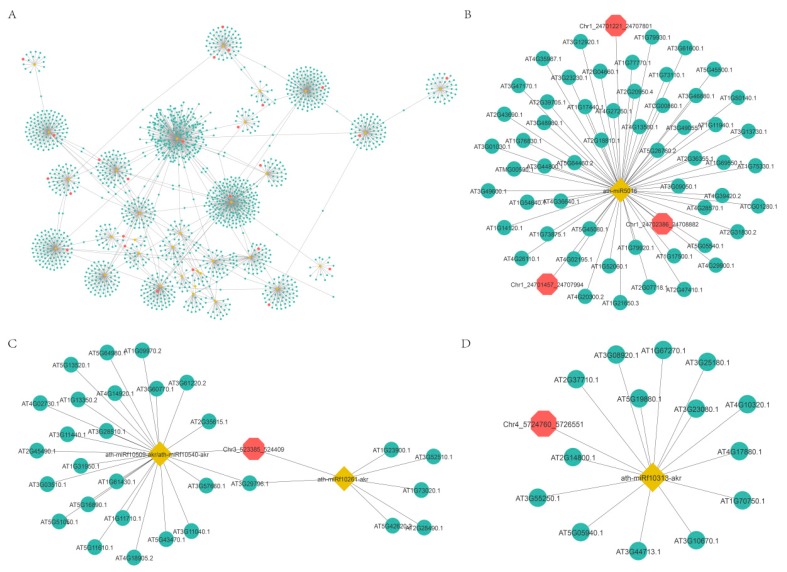
The circRNA-miRNA-mRNA interaction network. Yellow nodes: miRNAs. Red nodes: circRNAs that may be miRNA decoys. Green nodes: mRNAs that may be miRNA targets. (**B**–**D**) were extracted from (**A**).

Increasing evidence indicates that circRNAs might competitively bind to miRNAs and subsequently regulate their target genes by acting as miRNA decoys or sponges [49,50,51,52]. To further explore the functions of DEcircRNAs, we first predicted DEcircRNAs acting as miRNA decoys and mRNAs acting as miRNA targets. Then, a regulatory network of circRNA-miRNA-mRNA was constructed based on potential relationships between these RNAs (Figure 8A). This network was composed of 1698 nodes and 1780 edges, and the nodes included 32 miRNAs, 36 circRNAs (circRNAs acting as miRNA decoys), and 1630 mRNAs (mRNAs acting as miRNA targets) (Figure 8A and Table S3). CircRNAs had one to nine miRNA decoy sites (Figure 8A). For instance, circRNA Chr5:16093397|16096079 might act as a miRNA ath-miR864-5p decoy (Figure 8D). CircRNA Chr3:523385|524409 might act as a decoy for two miRNAs (ath-miRf10261-akr and miRNA ath-miRf10509-akr) (Figure 8C). In addition, multiple circRNAs might act as a decoy for one miRNA, such as circRNA Chr1:24701221|24707801, circRNA Chr1:24702386|24708882, and circRNA Chr1:24701457|24707994, all of which had miRNA ath-miR864-5p decoy sites (Figure 8B).

To further explore the functions of DEcircRNAs, 1630 mRNAs in the regulatory network of circRNA-miRNA-mRNA were subjected to GO analysis. We found that these mRNAs were mainly involved in plant growth and development process, response to various stimuli, response to JA, regulation of the JA-mediated signaling pathway, and JA metabolic process (Figure S1 and Table S4). These results indicated that these circRNAs in the circRNA-miRNA-mRNA network may play roles in JA-mediated signaling.

## 3. Discussion

CircRNAs are a special class of noncoding RNAs produced by non-linear backsplicing events between downstream splice donors and upstream splice acceptors. Recently, with the development of high-throughput sequencing technology, increasing numbers of circRNAs have been detected in plants and animals. In plants, circRNAs are closely related to plant development and stress responses, including biotic and abiotic stresses [20,22,23,24,25,26,27,28,30,53]. However, whether circRNAs participate in the pathways of plant responses to hormones is unclear. In this study, for the first time, circRNAs from *A. thaliana* seedlings at 24, 48, and 96 h after MeJA treatment were profiled. A total of 8588 circRNAs were detected in *A. thaliana* seedlings, of which 1526 circRNAs were predicted by CIRI2, 3410 by CIRCexplorer, and 4564 by find_circ. There was little overlap among the results of the different software programs (Figure S2). Software for the prediction of circRNAs is continually appearing, but different software programs have different advantages and shortcomings regarding sensitivity, precision, and computational cost. Such differences are largely due to the different strategies adopted [54,55,56]. CircRNAs predicted by all three methods accounted for a small proportion of the total predicted circRNAs, which indicated extensive differences among the prediction algorithms when they were applied to *A. thaliana* circRNA libraries. Thus, several tools should be used in combination to achieve reliable and comprehensive results. Consistent with previous studies, the circRNAs identified in this study were derived from exons, introns, and intergenic regions [13,15]. A large proportion of the circRNAs are derived from individual exons and may be related to the current mechanism of circRNA formation in plants: exon skipping events [34]. We found that although some genes produce more than one circRNA, most produce only one, which is consistent with previous reports in plants [56,57,58]. In addition, our results showed that many circRNAs were expressed at extremely low levels in *A. thaliana*. This characteristic may be a common basic feature of circRNAs in plants.

Phytohormones are important small signaling molecules in plants and play critical roles in various basic processes in plants [59]. MeJA is a key plant hormone and regulates pivotal processes, including seedling emergence, response to wounding, fertility, and growth-defense balance [60,61]. The biosynthesis, perception, transport, and signal transduction of MeJA have been extensively studied [59]. However, the roles of circRNAs in MeJA-mediated signaling pathways in *A. thaliana* have not been reported. Similar to other stresses in various plants, such as drought stress in maize and *A. thaliana* [20], dehydration stress in wheat [25], and cold stress in grapevine [28], MeJA treatment in *A. thaliana* altered the expression profiles of circRNAs. In this study, among the 8588 identified circRNAs, 385 circRNAs were identified as DEcircRNAs between MeJA-treated and untreated seedlings. The fluctuation of circRNA abundance after MeJA treatment in *A. thaliana* may be related to the possible roles of circRNAs in response to MeJA, such as some putative biomarkers of the JA response, or even some of them as potentials protein coding circRNAs that may be involved in JA signaling. In addition, nine circRNAs that were differentially expressed at three different time points between MeJA treated and controls plants may act as pivotal regulators in MeJA signaling, and the regulatory mechanisms warrant further study.

The circRNAs identified in many plants and animals, including human, have been reported to participate in the regulation of the expression of their host genes [11,12,13,15,32,34]. For example, some circRNAs regulate the expression of host genes by competing with canonical splicing [12]. In rice, overexpression of the circRNA “Os08circ16564” reduced the expression level of its host gene *AK064900* in both leaf and panicle tissues [21]. Recently, a circRNA from *SEPALLATA3* was shown to regulate splicing of its cognate mRNA through R-loop formation, resulting in transcriptional pausing [34]. In addition, some circRNAs in animals enhance transcription of their host genes by interacting with polymerase II or the U1 small nuclear ribonucleoprotein [11,62]. Therefore, in the present study, GO enrichment analysis of the host genes of DEcircRNAs was performed to investigate the functions of circRNAs in MeJA-mediated signaling. GO analysis showed that these host genes were mainly enriched in the categories cellular process, metabolic process, response to stimulus, and biological regulation. In addition, these host genes were associated with the GO terms developmental process, immune system process, reproduction, polysaccharide biosynthetic process, and response to reactive oxygen species; these findings are consistent with the reported functions of mRNAs that are differentially expressed after MeJA treatment [63], suggesting that the corresponding circRNAs may play important roles in regulating the expression of their host genes. In this study, we also performed expression pattern analyses of eight DEcircRNAs and their host genes and found that the expression patterns of two circRNAs were correlated with the expression patterns of their corresponding host genes, whereas those of the remaining six circRNAs exhibited no such correlations. The overexpression of the circRNA circR5g05160 in rice did not change the linear transcript accumulation of *LOC_Os05g05160* [63]. These data suggest that the regulatory effects of circRNAs on their host genes are diverse.

Previous studies have shown that circRNAs in animals and human can act as miRNA decoys or sponges to reduce the inhibition of miRNAs on their targets via circRNA-miRNA-mRNA networks [8]. For example, the circRNA hsa_circ_0005105 upregulates *NAMPT* expression via sponging miR-26a, thereby promoting extracellular matrix degradation of chondrocytes [10]. In addition, the circRNA circ_001350 regulates glioma cell proliferation, apoptosis, and metastatic properties by acting as a miRNA sponge [64]. In plants, circRNA-miRNA-mRNA networks have been identified, but they have not been confirmed by experiments [31,65]. To reveal whether DEcircRNAs can target miRNAs and participate in the transcriptional regulation of genes, we identified 385 DEcircRNAs predicted to contain miRNA decoy sites. As expected, 36 of the 385 DEcircRNAs were predicted to have one to nine miRNA decoy sites, which is consistent with other studies in plants [26,27,57]. For instance, circRNA Chr4:12480688|12481259 has nine miRNA decoy sites, indicating that it might regulate various processes through different miRNAs. In addition, three circRNAs (Chr1:24701221|24707801, Chr1:24702386|24708882, and Chr1:24701457|24707994) from the same host gene target the same miRNA, suggesting that plants regulate specific physiological processes through different forms of backsplicing. Moreover, by interacting with miRNAs, circRNAs can regulate the expression of miRNA target genes. In this study, GO enrichment analysis of the corresponding miRNA target genes was performed to identify genes that are regulated by circRNAs as miRNA decoys. These genes were enriched in the terms metabolic process, developmental process, RNA processing, protein phosphorylation, and DNA methylation or demethylation and may play important roles in MeJA-mediated signaling. In addition, we compared mRNAs in our network with transcriptomic data on the response to MeJA in *A. thaliana* from a previous study [63]; the network of mRNAs present in both our network and the previous study is displayed in Figure S4. These results suggest that some circRNAs may act as miRNA decoys to affect the expression of many genes involved in MeJA signaling. However, how these DEcircRNAs perform their functions by acting on their host genes or acting as miRNA sponges in the JA pathway is unclear and awaits further studies.

## 4. Materials and Methods

### 4.1. Plant Materials and Treatments

The *A. thaliana* ecotype Col-0 reserved in School of Life Sciences, Shaanxi Normal University was used in this study. Sterilized Col-0 seeds were cultured in 1/2 MS medium and placed in an incubator for two days at 4 °C in the dark for vernalization. Then, the seeds were germinated on 1/2 MS culture medium in an incubator for 6 days with a day/night (16 h/8 h) temperature of 23/16 °C. The seedlings were transferred to mock medium (comprising 9.17 μL of absolute ethanol added to 100 mL of 1/2 MS medium) and 10 μM of MeJA medium (created by diluting 2.5 μL of pure MeJA to 100 μL with absolute ethanol and adding 9.17 μL of diluted MeJA solution to 100 mL of 1/2 MS medium). Seedlings were transplanted into an incubator with a day/night (16/8 h) temperature of 23/16°C. Plant samples were collected at 24, 48, and 96h after transplantation.

### 4.2. RNA Extraction, cDNA Library Construction, and RNA Sequencing

After treating the seedlings with the control or MeJA medium, plant samples weighing at least 100 mg were collected, and total RNA was extracted using the RNAprep Pure Plant Kit (Tiangen, Beijing, China). The purity, concentration, and integrity of total RNA were detected by the Nanodrop, Qubit 2.0 and Agilent 2100 bioanalyzer respectively. A cDNA library was constructed using 1.5 μg total RNA according to the following steps: (a) rRNA was removed by rRNA probes (Ribo-ZeroTM rRNA Removal Kit, (Plant Leaf); Epicentre, Madison, WI, USA) and (b)linear RNA was removed by RNase R (Epicentre). Then, the rRNA in the remaining RNA was detected by PCR and gel electrophoresis. Next, the remaining RNAs were used to generate circRNA-seq libraries according to Zuo et al. [22]. The cDNA libraries were sequenced on an IlluminaHiSeq™ X-ten platform at Biomarker Technologies Co., Ltd. (Beijing, China), and 2 × 150 bp paired-end reads were obtained according to the standard Illumina protocol. The raw sequencing data were deposited in the US National Center for Biotechnology Information (NCBI) Sequence Read Archive under a Bioproject ID PRJNA597249.

### 4.3. CircRNA Identification and Differential Expression Analysis

Low-quality reads, including reads with greater than 50% unknown (N) bases or greater than 50% low-quality bases (Q ≤ 20), and adapters were removed from the sequencing data before circRNA identification by Trim galore (https://github.com/FelixKrueger/TrimGalore). The remaining clean reads were mapped to the *A. thaliana* reference genome TAIR10 using BWA (v0.7.17, mem-T 19) and Bowtie2 (v2.2.9) with default parameters [66,67]. The output of BWA was used to identify the circRNAs by CIRI (v2.0.6) with default parameters and CIRCexplorer with default parameters (v2.3.3), and the output of Bowtie2 was used to identify circRNAs by find_circ with default parameters(v1.0). One or more base differences may be present between the results of different prediction software programs. CD-HIT-EST (v4.6) was used to remove the repeated circRNAs in the prediction results based on the following inclusion criteria: (1) a length difference between the two sequences less than 10 bp and (2) an alignment sequence exceeding 99.7% of the shorter sequence. The other CD-HIT-EST parameters were the default parameters.

DEcircRNAs between the control- and MeJA-treated plants at the three time points were analyzed using the circMeta R package [68]. The results of Poisson-based test (*z*-test) were used to identify DEcircRNAs meeting the following criterion: false discovery rate (FDR) < 0.05. CircRNA expression levels were normalized to the RPKM value [number of circular reads/number of mapped reads (millions) × circRNA length (KB)]. Venn diagrams were generated using online tools (https://bioinfogp.cnb.csic.es/tools/venny/). A volcano plot and boxplot were constructed using the corresponding R packages.

### 4.4. Validation of circRNAs

Total RNA was extracted from *A. thaliana* seedlings at three time points using Plant RNA kit (Omega, Germany) according to the manufacturer’s protocol, and first-strand cDNA was synthesized from total RNA with random hexamer primers using the HiScript^®^II 1st Strand cDNA Synthesis Kit (+gDNA wiper) (Vazyme, Nanjing, China). To validate the existence of circRNAs, we designed convergent and divergent primers using GeneRunner software. PCR was performed using cDNA and gDNA as templates with two sets of primers. In addition, the PCR products were visualized by agarose gel electrophoresis and confirmed by Sanger sequencing. qRT-PCR was carried out with SYBR Green Master Mix (Vazyme, Nanjing, China) on a CFX 96 Real-Time PCR system (Bio-Rad, Hercules, CA, USA). The qRT-PCR procedure was as follows: 95 °C for 3 min, followed by 45 cycles of 95 °C for 30 s and 60 °C for 30 s. The 22^−∆∆*C*t^ method was used to calculate the relative expression of circRNAs [24,69], with GAPDH as the reference gene. The primers are listed in Table S5.

### 4.5. Functional Prediction of DEcircRNAs

The functions of some DEcircRNAs may depend on their host genes; thus, GO enrichment analysis of the host genes of the DEcircRNAs was performed using the online resource OmicShare (http://www.omicshare.com/).

In addition, the functions of some circRNAs may be independent of their host genes; they may act as miRNA decoys to achieve their effects [8]. To construct the networks among DEcircRNAs, miRNAs, and mRNAs, we obtained miRNA sequences from miRBase (http://mirbase.org/) and the plant microRNA database (PMRD: http://bioinformatics.cau.edu.cn/PMRD/) [70,71], and mRNA sequences were downloaded from Phytozome12 (https://phytozome.jgi.doe.gov/pz/portal.html). The DEcircRNAs sequences were extracted with an in-house Perl script. Next, GSTAr.pl (https://github.com/MikeAxtell/GSTAr) was used to establish the networks between circRNAs and miRNAs and between miRNAs and mRNAs, and the minimum free energy (MFE) of miRNA-circRNA or miRNA-mRNA duplexes was calculated with the RNAhybrid program. Then, the miRNA-targeted mRNA and miRNA-decoyed circRNA were predicted following a method proposed in a previous report [18,51,72]. The general criteria used to define a miRNA decoy were as follows: no more than six mismatched or inserted bases present between the ninth to 20th nucleotides of the miRNA 5’ end, perfect matching of the second to eighth bases of the miRNA 5’ end sequence, and no more than four mismatches or indels in other regions. Finally, the circRNA-miRNA-mRNA network was generated by Cytoscape (v3.7.2) [73].

## Supplementary Materials

Supplementary materials can be found at https://www.mdpi.com/1422-0067/21/3/792/s1. Table S1: Quality of the sequence data; Table S2: DEcircRNAs at the three time points; Table S3: DEcircRNAs acting as miRNA decoys; Table S4: GO enrichment analysis of mRNA in the circRNA-miRNA-mRNA network; Table S5: The primers used in this study; Table S6: GO enrichment analysis of the host genes of DEcircRNAs; Figure S1: GO enrichment analysis of mRNA involved in circRNA-miRNA-mRNA networks. (A) The most enriched GO terms of mRNA in circRNA-miRNA-mRNA networks. (B) Top 20 GO enrichment categories of mRNA in circRNA-miRNA-mRNA networks; Figure S2: The Venn diagram of the circRNAs were identified by three algorithms (CIRI2, CIRCexplorer, and find_circ); Figure S3: Un-modified gel photographs of five validated circRNAs. Note: The Sanger sequencing result for the third circRNAis incorrect. For each circRNA, the first column is a 2000bp marker, the second and third columns are gel results that using gDNA as a template, and the primers are divergent and convergent respectively; the fourth and fifth columns are gel results that using cDNA as a template, and the primers are divergent and convergent respectively; Figure S4: Network of mRNAs in both our network and a previous study. Yellow nodes: miRNAs. Red nodes: circRNAs that may be miRNA decoys. Green nodes: mRNAs that may be miRNA targets.
