# Supplementary figures and images for: Identification and Characterization of circRNAs Responsive to Methyl Jasmonate in Arabidopsis thaliana
Source: Int J Mol Sci. 2020 Jan 25;21(3):792. doi: 10.3390/ijms21030792 (PMC7037704; doi:10.3390/ijms21030792)

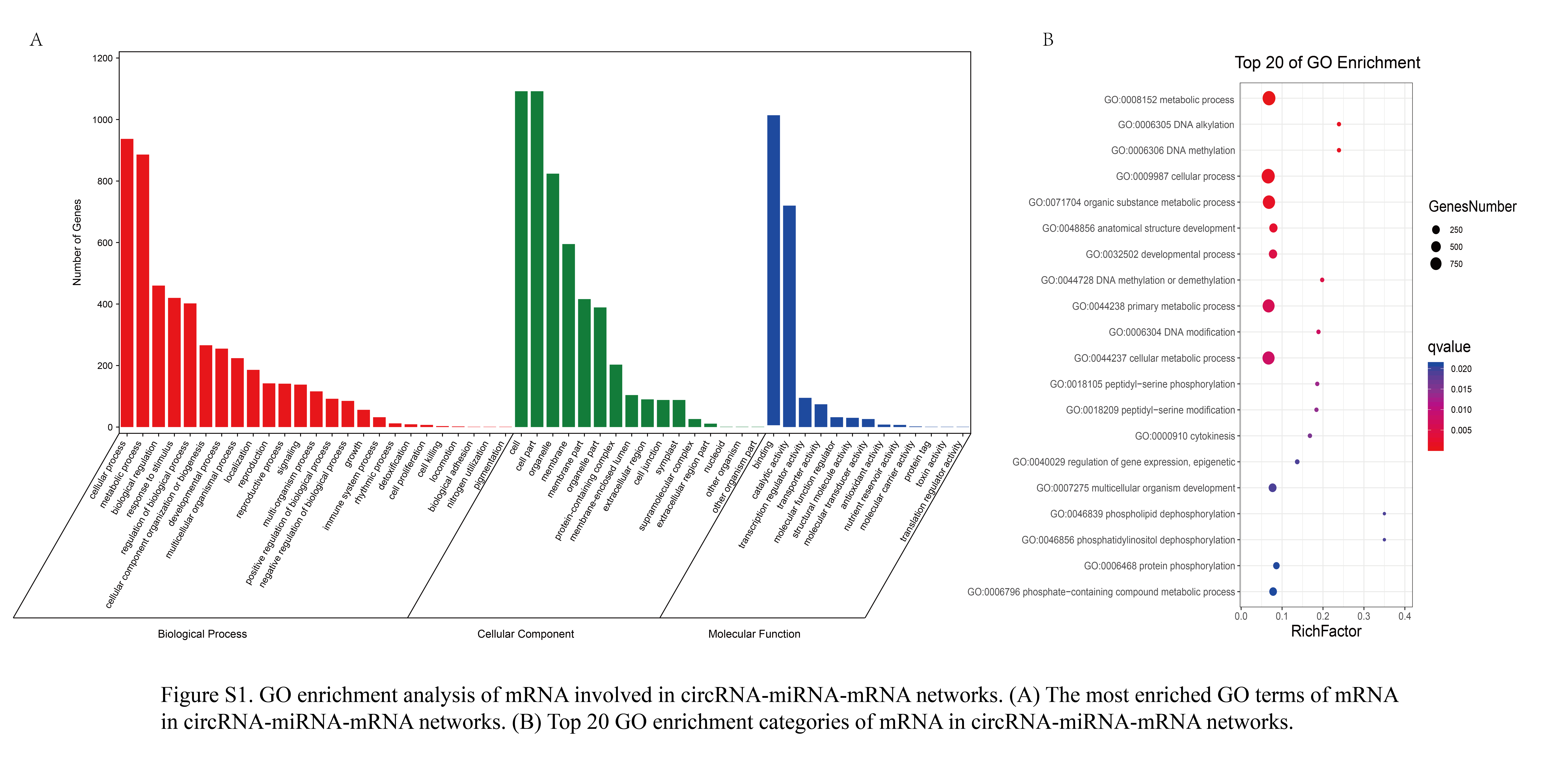

Supplement: Supplementary file 1 [file ijms-21-00792-s001.zip › Supplementary file/Figure S1.png]

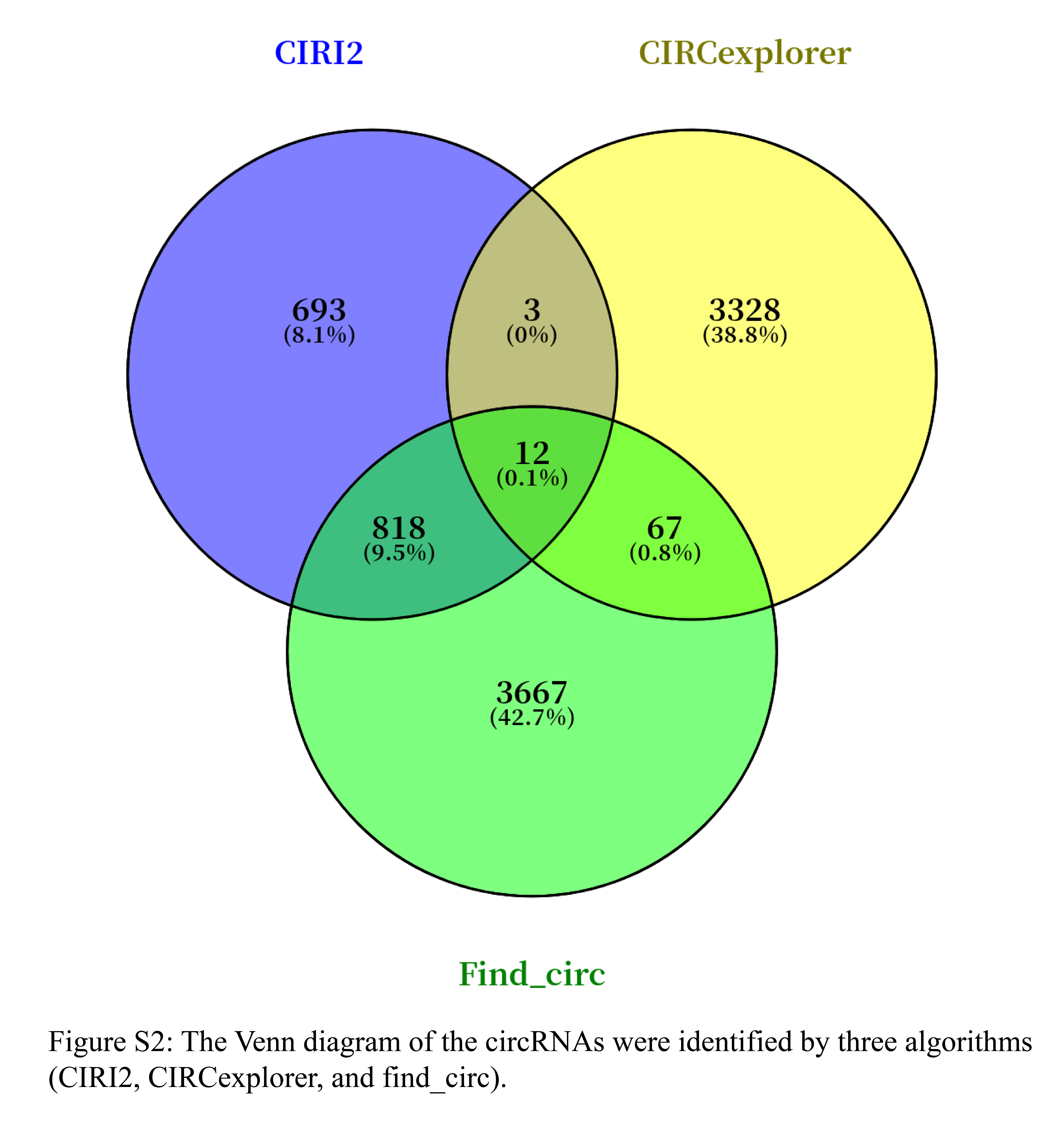

Supplement: Supplementary file 1 [file ijms-21-00792-s001.zip › Supplementary file/Figure S2.png]

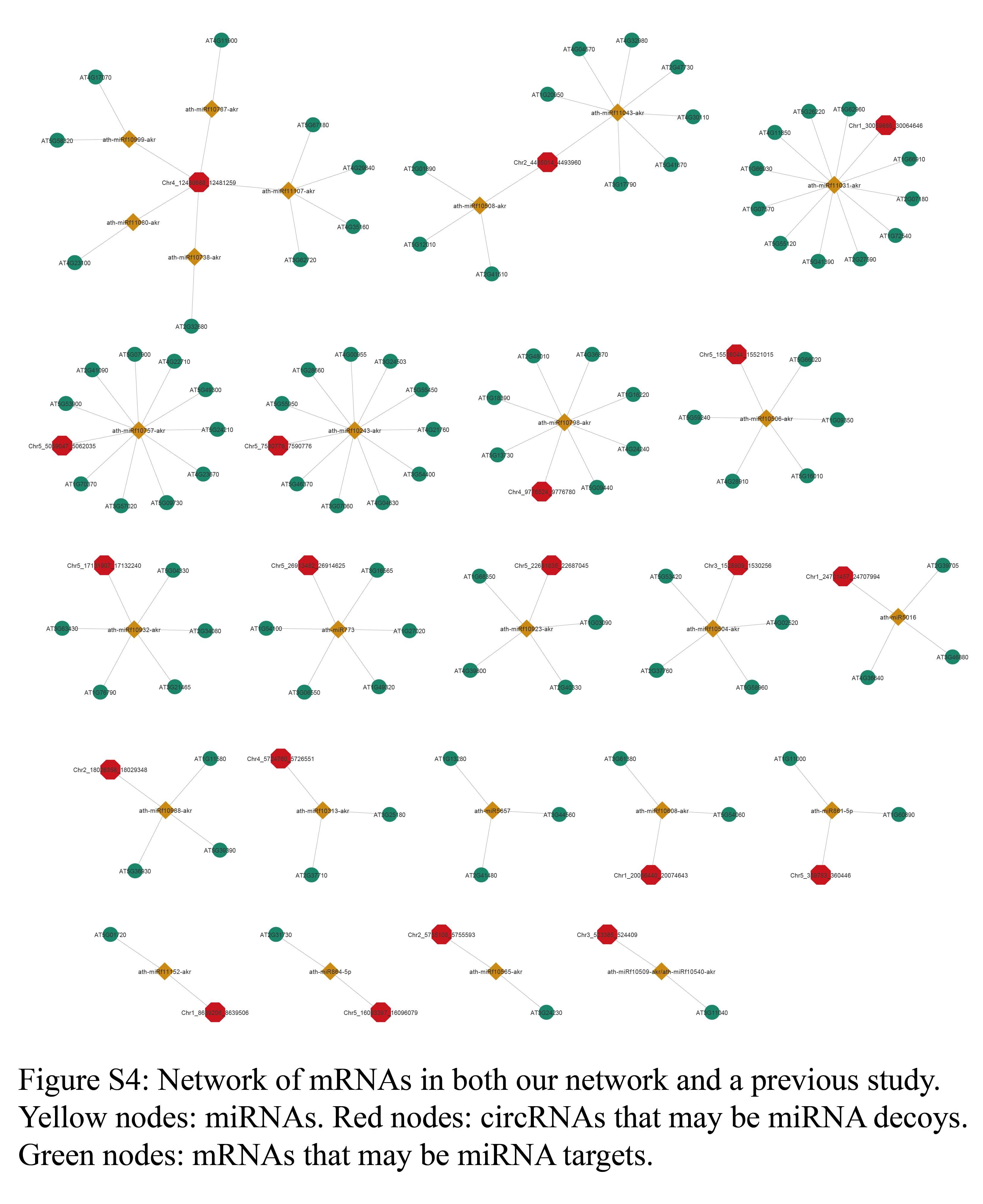

Supplement: Supplementary file 1 [file ijms-21-00792-s001.zip › Supplementary file/Figure S4.png]
